# Inducible cAMP Early Repressor Limits Glycolytic Programming in Regulatory T Cells and Supports Control of Inflammatory Colitis

**DOI:** 10.1002/eji.70297

**Published:** 2026-10-14

**Authors:** Aleksandra Lopez Krol, Luana Silva, Daniele C. Nascimento, Sophie M. Oberwalder, Hitikk Chawla, Gabriel Alberto Barbosa de Carvalho, Fatima Boukhallouk, Monica Romero, Anne Hausen, Matthias M. Gaida, Luciana Berod, José Carlos Alves‐Filho, Tim Sparwasser

**Affiliations:** ^1^ Institute of Medical Microbiology and Hygiene University Medical Center of the Johannes Gutenberg University Mainz Mainz Germany; ^2^ Center for Research in Inflammatory Diseases, Ribeirão Preto Medical School University of São Paulo Ribeirão Preto Brazil; ^3^ Institute of Pathology University Medical Center Mainz Mainz Germany; ^4^ Translational Oncology at the University Medical Center Mainz (TRON) Mainz Germany; ^5^ Research Center for Immunotherapy (FZI), University Medical Center Johannes Gutenberg‐University Mainz Mainz Germany; ^6^ Institute of Molecular Medicine University Medical Center of the Johannes Gutenberg‐University Mainz Mainz Germany

**Keywords:** cAMP metabolism, DSS colitis, glycolysis, ICER/CREM, regulatory T cells

## Abstract

Regulatory T cells (Tregs) are essential for immune tolerance, with cAMP metabolism central to their suppressive function. Inducible cAMP early repressor (ICER) is a negative regulator of cAMP signalling. However, its role in Treg biology is not fully understood. Here we generated Foxp3‐specific ICER knockout mice to assess ICER's function in Tregs. We found that ICER deficiency does not impair Treg development or suppression of effector T cell proliferation under homeostatic conditions. However, ICER‐deficient Tregs display enhanced Foxp3 stability, increased glycolysis and proliferation but exhibit impaired suppression of IFN‐γ production by effector T cells. In a dextran sodium sulphate‐induced colitis model, loss of ICER in Tregs led to aggravated intestinal inflammation and tissue damage, without, however, a marked effect on gross disease parameters. These results identify ICER as a context‐dependent modulator of Treg metabolism and inflammatory cytokine control, highlighting its potential role in regulating immune responses during inflammation.

## Graphical abstract

This study is focussed on the role of cAMP‐dependent transcriptional repressor ICER in regulatory T cell function. Using a conditional knockout model, Treg development, suppressive function, stability, and metabolic phenotype were examined to gain further understanding of the role of cAMP‐dependent signalling in regulatory T cells.

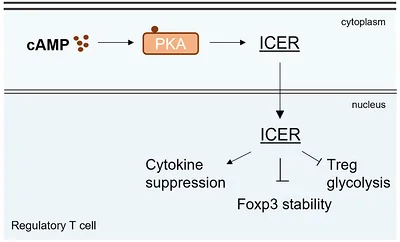

**Abbreviations**
ATF‐1: activating transcription factor 1
ATP: adenosine triphosphate
cAMP: cyclic adenosine monophosphate
CNS: conserved non‐coding sequence
CREB: cAMP‐response element‐binding protein
CREM: cAMP‐responsive modulator
DSS: dextran sodium sulphate
ECAR: extracellular acidification rate
EPAC1: exchange protein directly activated by cAMP 1
ICER: inducible cAMP early repressor
IFN: interferon
IL: interleukin
iTreg: induced Treg
LP: lamina propria
NFAT: nuclear factor of activated T cells
mLN: mesenteric lymph node
mTOR: mechanistic target of rapamycin
nTreg: natural Treg
OCR: oxygen consumption rate
OXPHOS: oxidative phosphorylation
PKA: protein kinase A
pTreg: peripheral Treg
ROS: reactive oxygen species
SD: standard deviation
SI: small intestine
SLO: secondary lymphoid organs
TCR: T cell receptor
Th: T helper cell
Treg: regulatory T cell
Teff: effector T cell

## 1 Introduction

Immune tolerance is maintained by Foxp3^+^ regulatory T cells (Tregs), which are generated either in the thymus as thymic or natural Tregs (tTregs or nTregs) or in the periphery from naïve T cells as peripheral Tregs (pTregs). These cells restrain inflammatory responses and protect the host from excessive or misdirected immune activity. The identity and function of Tregs are primarily defined by the transcription factor Foxp3, which is essential for their development and suppressive capacity. However, exposure to high levels of inflammatory cytokines such as IL‐6, IL‐4, or interferons (IFNs) can inhibit Foxp3 expression [1]. As a result, Tregs may lose their suppressive function and convert into so‐called ex‐Tregs, a population of former Tregs that can produce inflammatory cytokines and thereby promote, rather than suppress, inflammation [2]. This instability of the Treg lineage is central to many autoinflammatory pathologies, highlighting the importance of understanding the mechanisms that preserve Treg function for the development of new therapeutic strategies [3].

T cell effector function is closely interlinked with metabolic state, making it susceptible to metabolic perturbations [4, 5]. Upon TCR stimulation, T cells undergo metabolic adaptation, including increased glycolysis to support rapid proliferation [6]. Although this shift occurs across T cell subsets, metabolic requirements differ according to lineage and function [7]. In Tregs, Foxp3 promotes mitochondrial metabolism and oxidative phosphorylation (OXPHOS), thereby supporting suppressive function and lineage stability [8, 9, 10, 11]. At the same time, Tregs engage glycolytic programs to support proliferation and migration [6, 12]. Thus, distinct Treg functions appear to rely on partially separable metabolic modules rather than a single dominant metabolic state [3].

Another feature of Tregs, crucial for their suppressive function, is their elevated intracellular concentration of cyclic adenosine monophosphate (cAMP) [13, 14]. cAMP is generated from ATP by adenylyl cyclases in response to activation of G‐protein‐coupled receptors by signals such as chemokines or hormones. cAMP signalling operates through both the canonical protein kinase A (PKA) and the non‐canonical exchange protein directly activated by cAMP 1 (EPAC1) pathway [15, 16]. Among the most prominent PKA‐regulated targets in T cells are transcription factors of the CREB/CREM/ATF‐1 family, including cAMP‐response element‐binding protein (CREB) and cAMP‐responsive modulator (CREM), which regulate T cell activation [17, 18]. Transcription factors of this family function as homo‐ and heterodimers to regulate or promote transcription by binding to specific cAMP response elements (CREs) in gene promoters [17, 18]. Inducible cAMP early repressor (ICER) is a truncated isoform of CREM, originating from the P2 promoter within the *Crem* gene, and consists only of the DNA‐binding domain and lacks the transactivation domain of other family members. ICER can form heterodimers with other CREB/CREM/ATF‐1 family members, thereby inhibiting their function [17, 19, 20]. This provides a negative feedback mechanism for cAMP‐mediated signalling. A role for ICER in immune regulation is known in T effector cells (Teffs). There, ICER translocates to the nucleus upon exogenous cAMP stimulation, where it interferes with the activation of the IL‐2 promoter by the transcription factor NFATc1 [21].

Interestingly, ICER has also been detected in the nucleus of nTregs [21]. In contrast to Teff cells, where nuclear translocation of ICER requires exogenous cAMP stimulation, nTregs display nuclear ICER upon TCR stimulation alone, likely due to their constitutively elevated intracellular cAMP levels. Given the central role of cAMP in Treg identity, it remains unclear whether ICER is necessary for Treg development, lineage stability, suppressive function, or metabolic adaptation under inflammatory conditions. To address this, we generated a conditional ICER knockout model and tested whether ICER regulates Treg stability, metabolism, and effector control under homeostatic and inflammatory conditions.

## 2 Results

### 2.1 Deletion of ICER Does Not Impair Treg Development or Suppressive Function Under Homeostatic Conditions

To investigate the role of ICER in Treg function, we crossed *ICER* ^fl/fl^ mice [22] with *Foxp3* ^cre^ mice [23] to generate the Treg‐specific ICER‐deficient line *ICER* ^fl/fl^ *Foxp3* ^cre^. Efficient loss of the P2‐derived ICER transcript in Tregs was confirmed by RT‐qPCR (Figure S1A). A germline‐recombined ICER allele arose during breeding and was subsequently maintained as the ICER ko allele. Germline recombination of floxed alleles has previously been reported for several Cre‐driver lines, including the Rgs9 Cre line [24]. The ICER ko allele used here was validated by locus‐specific genotyping and then used as the *ICER* ^ko/ko^ line to examine ICER function independently of Foxp3 (Figure S1A). To determine whether the loss of ICER affects Treg development, tissue distribution, or survival under homeostatic conditions, we analysed Treg frequencies and absolute numbers in secondary lymphoid organs (SLOs) and in the small intestinal lamina propria (LP) of *ICER* ^fl/fl^ *Foxp3* ^cre^ mice in comparison with *ICER* ^fl/wt^ *Foxp3* ^cre^ and *ICER* ^wt/wt^ *Foxp3* ^cre^ littermates. No significant decrease in Foxp3^+^ cell frequencies was observed, and total splenic Foxp3^+^ cell numbers were increased (Figure 1A; Figure S1B,C). When we assessed Treg markers CTLA‐4, Helios, and CD39, no differences could be detected (Figure S1D–G). Consistently, *in vitro* iTreg differentiation assays using *ICER* ^ko/ko^ mice, together with analysis of Foxp3^+^ cell frequencies in SLOs of *ICER* ^ko/ko^ mice, indicated that the loss of ICER does not impair Treg development in either the thymus or the periphery (Figure S1H–J).

**FIGURE 1:**
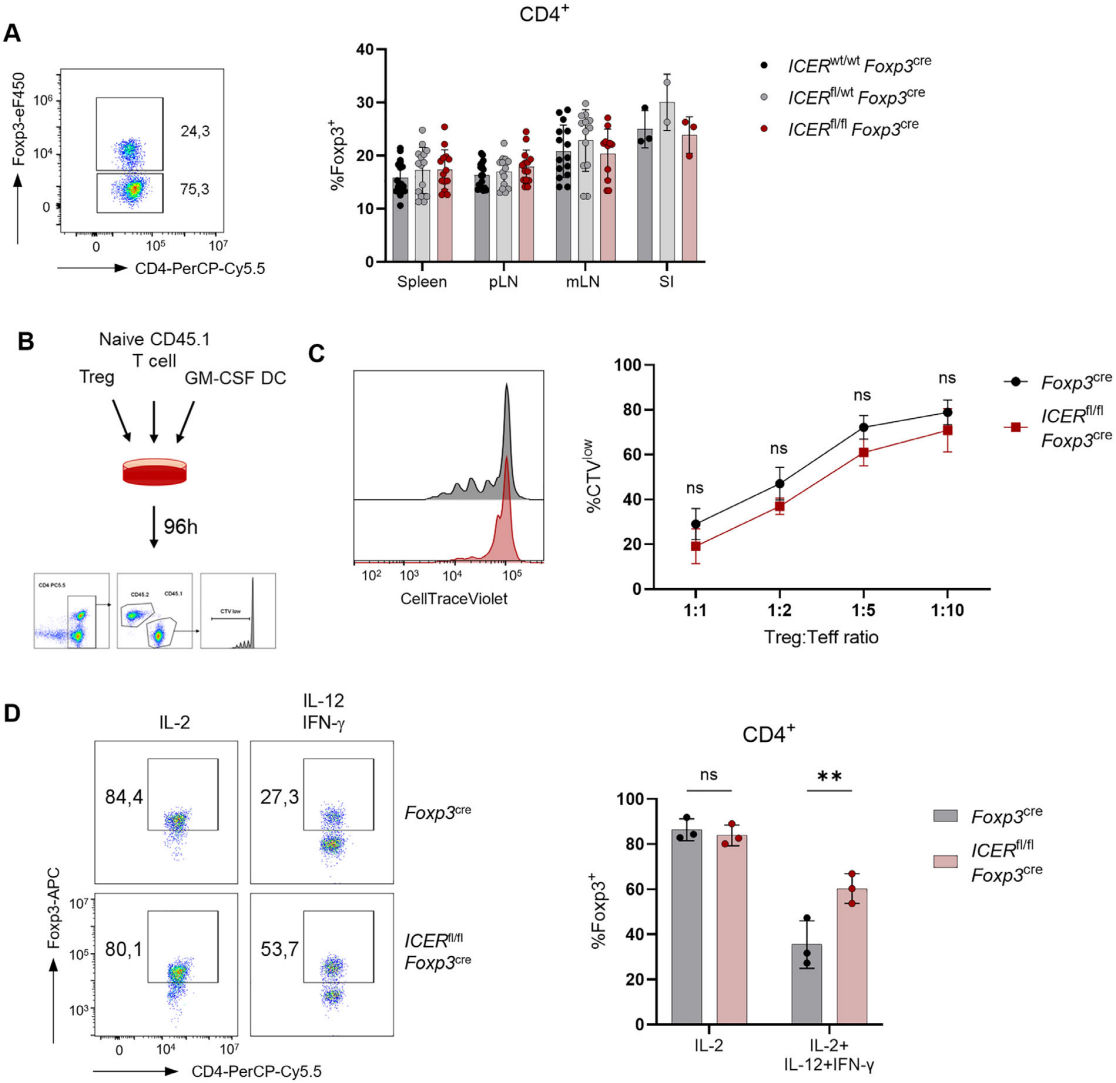
ICER is dispensable for Treg differentiation and suppression of Teff proliferation, but limits maintainance of Foxp3 expression. (A) SLOs and LP of the small intestine of *ICER* ^fl/fl^ *Foxp3* ^cre^, *ICER* ^fl/wt^ *Foxp3* ^cre^, and *ICER* ^wt/wt^ *Foxp3* ^cre^ mice were analysed by flow cytometry. (B) CD45.1^+^ T cells were co‐incubated with DCs and CD45.2^+^ WT or ICER‐deficient Tregs for 96 h, and proliferation was assessed by flow cytometry. (C) Percentages of proliferating (CTV^low^) CD45.1^+^ cells after 96 h of incubation depending on Treg:Teff ratio and Treg genotype (right). Representative histogram overlay of CTV in CD45.1^+^ T cells (left). (D) Sorted Tregs from SLOs of *Foxp3* ^cre^ and *ICER* ^fl/fl^ *Foxp3* ^cre^ mice were expanded for 12 days with and without the presence of IL‐12 and IFN‐γ. Representative flow cytometry plot of Foxp3 (left) and Foxp3 percentages depending on the presence of IL‐12/IFN‐γ and Treg genotype (right). Dots represent biological replicates. Two‐way ANOVA with multiple comparisons was used for statistical analysis. Mean ± SD, *p < 0.05, **p < 0.01, ***p < 0.001, ns, not significant.

Because cAMP is central to Treg‐mediated suppression of Teff proliferation, we next investigated whether the loss of ICER affects Treg suppressive function. We performed *in vitro* suppression assays in which sorted nTregs from SLOs of *ICER* ^fl/fl^ *Foxp3* ^cre^ and *Foxp3* ^cre^ mice were co‐cultured with naïve CD45.1^+^ T cells and dendritic cells. After 96 h, Teff proliferation was assessed (Figure 1B; Figure S2A). We found that suppression of Teff proliferation was independent of ICER expression in Tregs (Figure 1C). To further test the role of ICER under conditions of enhanced cAMP signalling, we performed suppression assays in the presence of NECA, a broadly acting adenosine receptor agonist that increases cAMP signalling [25]. While NECA treatment induced *Icer* transcription (Figure S2B), it also increased suppression by both WT and ICER‐deficient Tregs (Figure S2C,D), suggesting that cAMP‐mediated suppression of Teff proliferation by Tregs does not require ICER.

### 2.2 Lack of ICER Improves Foxp3 Stability in a Th1‐Like Inflammatory Environment

As cAMP metabolism has been linked to Treg stability, we next asked whether ICER contributes to the maintenance of Foxp3 expression under inflammatory conditions. It is well‐established that exposure to inflammatory cytokines such as IFN‐γ can destabilise Foxp3 expression in Tregs, which impacts their function and contributes to autoinflammatory diseases [1]. To address this, we cultured sorted Tregs from SLOs of *ICER* ^fl/fl^ *Foxp3* ^cre^ and *Foxp3* ^cre^ mice for up to 12 days either under control conditions (IL‐2) or in the additional presence of IL‐12 and IFN‐γ. Surprisingly, ICER deficiency significantly improved Treg stability in the presence of IL‐12 and IFN‐γ, although no difference was observed under control conditions (Figure 1D). We tested Th1 differentiation in the absence of ICER to understand whether the general IL‐12 response could be compromised, but found comparable differentiation in WT and ICER‐KO cells (Figure S2E). To determine whether the increased stability of ICER‐deficient Tregs translated to enhanced suppressive activity, we next performed suppression assays under inflammatory conditions. For this, iTregs were generated from *ICER* ^fl/fl^ *Foxp3* ^cre^ and *Foxp3* ^cre^ mice (Figure 2A), and IL‐12 was added to suppression assay co‐cultures. Although the overall suppressive capacity was modestly reduced in the presence of IL‐12, no significant difference in suppression of Teff proliferation was observed between ICER‐competent and ICER‐deficient Tregs (Figure 2B).

**FIGURE 2:**
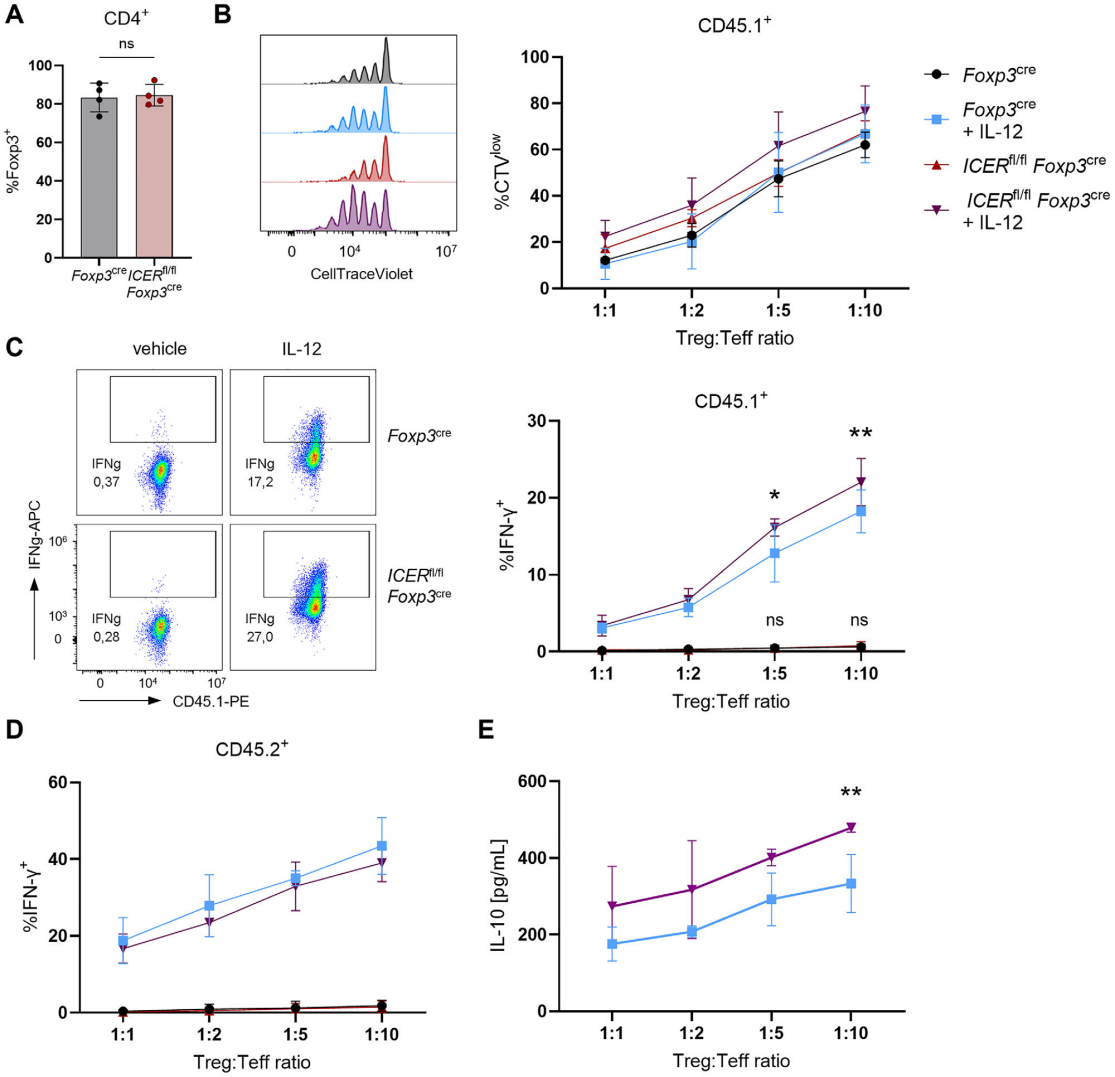
ICER‐deficient Tregs fail to control IFN‐γ production by effector T cells. (A) Sorted naïve T cells from *Foxp3* ^cre^ or *ICER* ^fl/fl^ *Foxp3* ^cre^ mice were expanded in iTreg polarising conditions for 96 h. (B) Percentages of proliferating (CTV^low^) CD45.1^+^ cells after 96 h of incubation depending on Treg:Teff ratio and Treg genotype. Representative histogram overlay of CTV of CD45.1^+^ T cells (left) and CTV^low^ percentages depending on Treg:Teff ratio, Treg genotype, and treatment (right). (C) IFN‐γ production of CD45.1^+^ cells was assessed after 96 h of co‐culture by flow cytometry. Representative flow cytometry plots (left) and percentages of IFN‐γproduction by CD45.1^+^ T cells depending on Treg:Teff ratio, Treg genotype, and presence of IL‐12 (right). Statistical comparison between genotypes., (D) IFN‐ γ production of CD45.2^+^ cells was assessed after 96 h of co‐culture by flow cytometry. Percentages of IFN‐γproduction by CD45.2^+^ Tregs depending on Treg:Teff ratio, Treg genotype, and presence of IL‐12. (E) ELISA measurement of IL‐10 concentration in supernatant after 96 h of suppression assay depending on Treg:Teff ratio, Treg genotype and presence of IL‐12. Dots represent biological replicates. (B–E) Three biological replicates each. Two‐way ANOVA with multiple comparisons was used for statistical analysis. Mean ± SD, *p < 0.05, **p < 0.01, ***p < 0.001, ns, not significant.

In addition to suppressing proliferation, Tregs are also known to restrain inflammatory cytokine production by Teff cells [26]. We therefore assessed IFN‐γ production by CD45.1^+^ Teff cells after 96 h of co‐culture. CD45.1^+^ Teff‐cell IFN‐γ production was higher in co‐cultures containing ICER‐deficient Tregs than WT Tregs, particularly at low Treg:Teff ratios (Figure 2C). This difference did not appear to originate from the Treg‐intrinsic IFN‐γ production, since upregulation of IFN‐γ in response to IL‐12 stimulation was not altered in the absence of ICER (Figure 2D).

Because IL‐10 was previously shown to be involved in the regulation of IFN‐γ production [27], we next used ELISA to measure IL‐10 concentrations in suppression assay supernatants after 96 h of co‐culture. Surprisingly, we observed an increase in IL‐10 levels in the supernatants from ICER‐deficient Treg co‐cultures relative to co‐cultures containing WT Tregs (Figure 2E). These data reveal a dissociation between Foxp3 stability and inflammatory effector control. While ICER limits Foxp3 stability, it supports the ability of Tregs to restrain inflammatory cytokine production by Teff cells.

### 2.3 ICER Restrains Treg Glycolysis

Treg proliferation is known to be supported by glycolytic metabolism [6]. We observed that ICER‐deficient Tregs were more abundant in the spleen (Figure S1B) and, consistently, sorted ICER‐deficient nTregs proliferated more strongly during IL‐2‐driven expansion, without a reduction in cell survival (Figure 3A; Figure S3A–C). To investigate the metabolic basis of this enhanced proliferation, we performed Seahorse analysis of iTregs to assess their metabolic status. ICER‐deficient iTregs displayed significantly increased basal glycolysis and glycolytic capacity (Figures 3B,C), consistent with their enhanced proliferative behaviour. Additionally, we examined mitochondrial metabolism and found that maximal respiration was significantly increased in ICER‐deficient cells, whereas basal respiration was unchanged (Figure S3D,E). We also observed a reduced OCR/ECAR ratio in ICER‐deficient Tregs (Figure 3D), suggesting a relative shift toward glycolytic metabolism. Interestingly, increased glycolytic activity did not confer a survival disadvantage for ICER‐deficient Tregs in low glucose conditions (Figure S3F). Since mTOR signalling is a central regulator of glycolytic programming, we wondered whether altered mTOR activity could account for this phenotype. Unexpectedly, Western blot analysis of mTOR phosphorylation at Ser2448 did not reveal any difference between WT and ICER‐deficient iTregs (Figure 3E; Figure S3G). To further investigate whether changes to mTOR activity accounted for the increased glycolysis in ICER‐deficient cells, we added 100 nM rapamycin to iTreg cultures after 72 h of differentiation and performed Seahorse analysis 24 h later. Addition of Rapamycin decreased glycolytic capacity in both ICER‐competent and ICER‐deficient cells (Figure 3F; Figure S3H). However, glycolysis levels of ICER‐deficient Tregs remained higher than in WT Tregs, with glycolytic capacity still showing a significant increase, indicating an additional mTOR‐independent regulatory mechanism (Figure 3G). Enhanced mTOR signalling is known to impair Treg suppressive function and thus, the absence of an effect upon mTOR phosphorylation in ICER‐deficient Tregs is consistent with their unchanged suppressive capacity (Figure 1C). Together, these data suggest that increased glycolysis in ICER‐deficient Tregs is not fully normalised by rapamycin and is therefore not fully accounted for by the measured mTOR axis.

**FIGURE 3:**
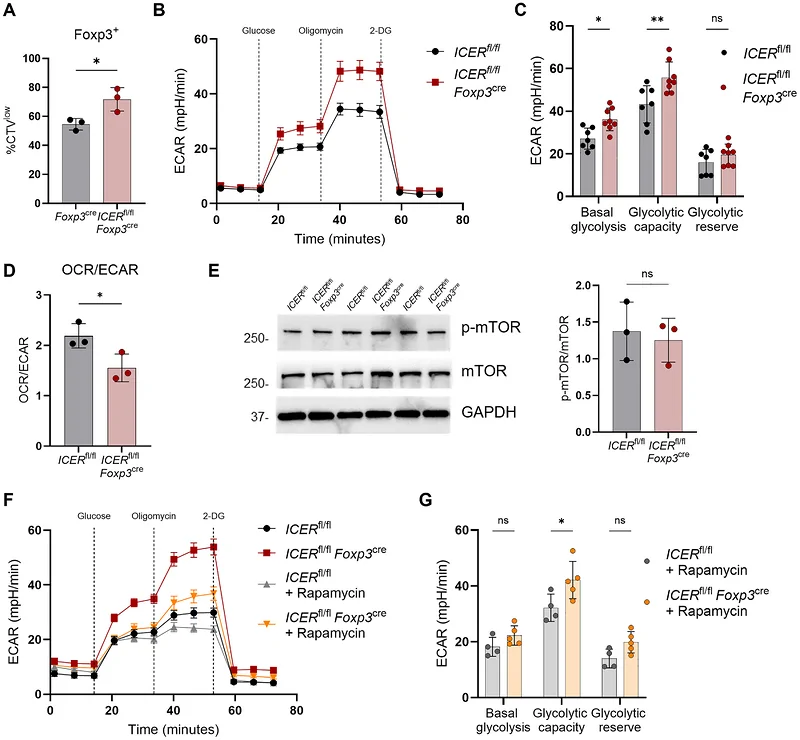
ICER regulates glycolysis in Tregs. (A) Sorted Tregs from SLOs of *Foxp3* ^cre^ and *ICER* ^fl/fl^ *Foxp3* ^cre^ mice were stained with CTV, expanded for 96 h, and stained for flow cytometry analysis. (B) Sorted naive T cells from *ICER* ^fl/fl^ and *ICER* ^fl/fl^ *Foxp3* ^cre^ mice were expanded in iTreg‐polarising conditions for 96 h and extracellular acidification rate (ECAR) was assessed by Seahorse analysis. (C) Quantification of basal glycolysis, glycolytic capacity, and glycolytic reserve. (D) OCR/ECAR ratio was determined from basal respiration and glycolysis rates. (E) Western blot analysis of mTOR phosphorylation in WT and ICER‐deficient iTregs after 96 h of differentiation (left). Quantification (right). (F) Sorted naive T cells from *ICER* ^fl/fl^ and *ICER* ^fl/fl^ *Foxp3* ^cre^ mice were expanded in iTreg‐polarising conditions for 96 h, 100 nM Rapamycin was added after 72 h, and the extracellular acidification rate (ECAR) was assessed by Seahorse analysis. (G) Quantification of basal glycolysis, glycolytic capacity, and glycolytic reserve. Student's *t*‐test (A, D) and Two‐way ANOVA with multiple comparisons (C, G) were used for statistical analysis. Dots represent biological replicates. Mean ± SD, *p < 0.05, **p < 0.01, ***p < 0.001, ns, not significant.

### 2.4 Deletion of ICER in Tregs Exacerbates Pathology During DSS Colitis

Finally, to test our findings *in vivo*, we performed dextran sodium sulphate (DSS) colitis experiments on *ICER* ^fl/fl^ *Foxp3* ^cre^ animals and *ICER* ^fl/fl^ littermates (Figure 4A). The general disease parameters, such as body weight or colon shortening, were not markedly different between the groups (Figure 4B; Figure S4A). Histological analysis of colon sections, however, revealed more severe inflammation, greater inflammatory extent, more pronounced epithelial alterations by trend, and significantly increased disruption of mucosal architecture in *ICER* ^fl/fl^ *Foxp3* ^cre^ mice (Figures 4C,D). These findings prompted us to investigate whether Tregs in the inflamed intestinal environment could fail to adequately control effector cytokine responses. Analysis of Tregs from the mesenteric lymph nodes (mLNs) and colonic LP revealed a modest increase in Treg frequencies and absolute numbers in both compartments, as well as a non‐significant trend towards higher IL‐10 frequencies consistent with increased Treg accumulation and/or maintenance in inflamed tissue (Figure 4E; Figure S4B–D). Further characterisation of the Treg compartment showed that neither RORγt expression nor cytokine production by Tregs nor Treg markers CTLA‐4, CD39, Helios and ST‐2 were significantly altered by ICER deficiency (Figure 4F,G; Figure S4E–J). Similarly, there was no increase in RORγt‐expressing or IL‐17A‐producing Foxp3^−^ CD4^+^ T cells, indicating no shift in Treg/Th17 balance (Figure 4H; Figure S4K), and no increase in TNF‐α production (Figure S4L). In contrast, and in line with our *in vitro* data, we found a significant increase in IFN‐γ^+^ Teff cells in colonic LP, indicating that ICER expression in Tregs contributes to control of inflammatory IFN‐γ production by Teff cells (Figure 4I). TIGIT‐expressing Tregs are known to suppress Th1 and Th17 responses and promote tissue repair [28, 29]. When we analysed TIGIT expression, we unexpectedly found that in ICER‐deficient mice, a higher percentage of Tregs expressed TIGIT (Figure 4J). This raises the possibility that in ICER‐deficient Tregs IFN‐γ‐control and tissue‐repair functions are impaired while TIGIT expression is retained, fitting our observation of increased tissue damage in *ICER* ^fl/fl^ *Foxp3* ^cre^ mice.

**FIGURE 4:**
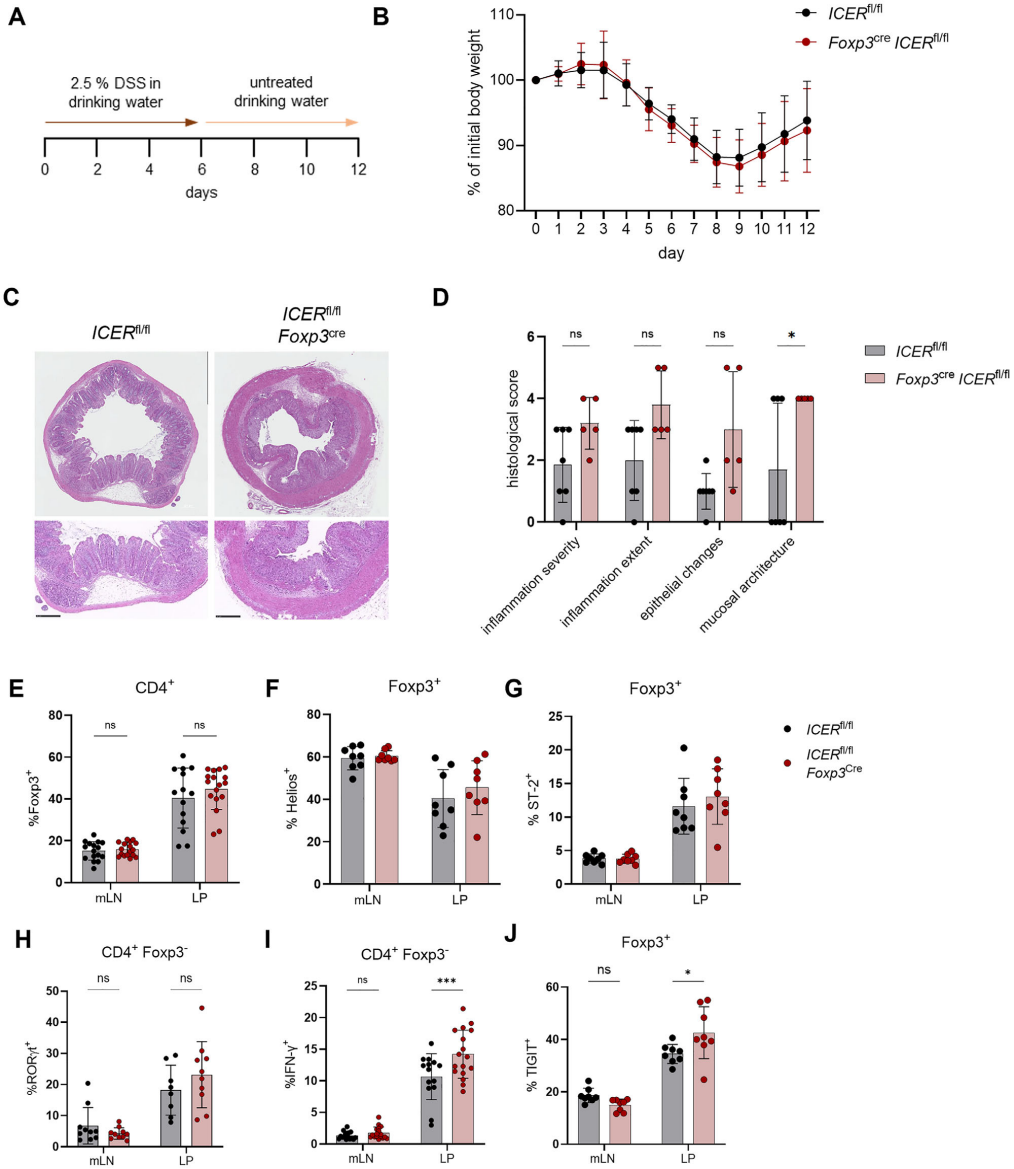
ICER deficiency in Tregs exacerbates histopathology during DSS colitis. (A) Experimental design. *ICER* ^fl/fl^ *Foxp3* ^cre^ and *ICER* ^fl/fl^ littermates were treated for 6 consecutive days with 2.5% DSS in the drinking water, followed by 6 days of untreated water. (B) Weight was assessed daily, and body weight loss was calculated. (C) Representative H&E‐stained colon sections on day 12. Scale bar: 250 µm for the detailed section and 200 µm for the overview section. (D) Histological scores. (E–G) Flow‐cytometric analysis of mesenteric lymph nodes and colonic lamina propria on day 12. (E) Frequencies of Foxp3^+^ cells among CD4^+^ cells. (F) Frequencies of Helios^+^ cells among Foxp3^+^ cells. (G) Frequencies of ST‐2^+^ cells among Foxp3^+^ cells. (H) Frequencies of RORγt^+^ cells among CD4^+^ Foxp3^−^ cells. (I) Frequencies of IFN‐ γ^+^ cells among CD4^+^ Foxp3^−^ cells. (J) Frequencies of TIGIT^+^ cells among Foxp3^+^ cells. Dots represent biological replicates. Error bars represent SD. For (B), repeated‐measures two‐way ANOVA and for (D–J), two‐way ANOVA with multiple comparisons were used for statistical analysis. Mean ± SD,490846870 CRC/TRR 355/1 B08 *p < 0.05, **p < 0.01, ***p < 0.001, ns, not significant.

Together, these findings suggest that ICER in Tregs contributes to control of inflammatory effector responses *in vivo*, consistent with ICER‐mediated regulation of Treg metabolic and suppressive function.

## 3 Discussion

ICER has long been proposed to contribute to Treg function, yet its precise role in Treg biology has remained unclear [21, 30]. Tregs are characterised by elevated intracellular cAMP levels [13], and considerable effort has been devoted to understanding how cAMP signalling contributes not only to suppression of effector T cells, but also to Treg‐intrinsic physiology. Previous work has established an important role for cAMP signalling in Treg function, including contributions of the non‐canonical EPAC1 pathway to suppressive activity [31]. However, the role of ICER in Tregs is likely mechanistically distinct, as ICER is induced downstream of the classical PKA axis rather than EPAC1 [31, 32]. Our data identify ICER as a context‐dependent regulator of Treg biology that, while dispensable for Treg development and homeostatic suppressive function, influences maintenance of Foxp3 expression and control of inflammatory IFN‐γ responses. One of the most notable findings of our study is that loss of ICER improved Foxp3 stability under inflammatory conditions. Because we did not detect defects in either thymic or peripheral Treg development, the effects of ICER appear to relate primarily to maintenance of lineage stability rather than lineage specification. This is consistent with the role of the Foxp3 locus in integrating environmental cues that shape the stability of the Treg program [33]. Among the conserved non‐coding sequences at the Foxp3 locus, CNS2 is particularly important for stable maintenance of Foxp3 expression [34]. Because ICER affected Foxp3 stability rather than thymic or peripheral Treg development, one possible explanation is that it influences transcription factor access to the Foxp3 CNS2 region. Given that CREB, a known target of ICER‐mediated transcriptional interference, has been implicated in stabilising Foxp3 expression through binding to demethylated CNS2 [35], it is plausible that loss of ICER enhances CREB‐dependent support of the Foxp3 locus. Future studies will need to directly assess CREB occupancy, DNA methylation status, and chromatin accessibility at CNS2. Thus, ICER appears to act as a negative regulator of Foxp3 maintenance under inflammatory conditions, although the underlying mechanism remains to be defined.

We observed increased IL‐10 concentrations in co‐culture supernatants in the presence of ICER‐deficient Tregs. However, the cellular source of IL‐10 was not directly resolved. Previous studies have implicated the CREB/ATF1 transcriptional axis, including ICER, in the regulation of *IL‐10* expression in other cell types [36], and recent work has shown that CREB deficiency in Tregs can be associated with increased IL‐10 production [37]. In our system, these findings are compatible with a role for ICER in modulating IL‐10‐associated transcriptional programs, but the underlying mechanism remains unresolved.

Increased glycolysis rates are known to correlate with stronger Treg proliferation and decreased stability and suppressive function [8, 12, 38]. The most ubiquitous regulator of T cell metabolism and function is mTOR, and its increased activity often underlies increased glycolysis and diminished OXPHOS [39]. We show that ICER deficiency results in strongly increased glycolytic activity and proliferation in Tregswithout a detectable effect on mTOR phosphorylation or decrease in OXPHOS levels and suppression of Teff proliferation. These data suggest that ICER may restrain glycolysis in Tregs through a mechanism not explained by the measured mTOR phosphorylation readout. One of the factors controlling glycolysis rate is glucose influx, carried out by several glucose transporters [11, 40]. In addition to well‐known Glut1, Glut3 has recently emerged as an important glucose transporter in Tregs [41]. *Slc2a3* expression was shown to be induced by CREB [42], which raises the hypothesis that ICER may restrain Glut3 expression, providing a possible mechanism for an mTOR‐independent regulation of glycolysis.

A link between glycolysis and cytokine regulation was suggested previously due to an observation that Tregs lacking PTEN upregulate glycolytic metabolism through mTOR and fail to suppress IFN‐γ production in Teffs [9]. ICER deficiency impaired control of IFN‐γ production by Teff cells *in vitro* and *in vivo*. Because ICER‐deficient Tregs also showed increased glycolysis, these findings raise the possibility of a link between metabolic state and cytokine‐control function.

The Treg/Th17 balance influences the severity of intestinal inflammation during colitis. Therefore, modulation of Treg suppressor function or stability can impact disease outcome. We did not detect reduced Treg frequency or overt evidence of destabilisation, and no increase in Th17 cells characterised by RORγt expression and IL‐17 production was observed. Nevertheless, gut inflammation was exacerbated in *ICER* ^fl/fl^ *Foxp3* ^cre^ mice. IFN‐γ is central to gut inflammation during DSS colitis [43]. We found that ICER‐deficient Tregs were associated with increased Teff‐cell IFN‐γ production and exacerbated histopathology. Through CREB, a transcription factor antagonized by ICER, cAMP signalling has been shown to promote Th1 differentiation by inducing gene expression of IL‐12 and IFN‐γ receptors [44]. A similar role for CREB in Tregs could contribute to a stronger loss of cytokine suppressive capacity by ICER‐deficient Tregs in the presence of IL‐12, as observed in this study. Additionally, deletion of CREB in Tregs has recently been shown to increase Treg fitness and suppression of gut inflammation [37]. Therefore, it is possible that Treg cytokine suppression is attenuated through loss of ICER‐mediated inhibition of CREB signalling. In a colitis setting, cAMP signalling via PKA in response to butyrate has been shown to promote intestinal Tregs through modulation of mTOR [45]. While we did observe comparable Foxp3 frequencies in the absence of ICER in DSS colitis, suppression was not improved, in agreement with our finding that we did not observe differences in mTOR Ser2448 phosphorylation dependent on ICER. Recently, TIGIT^+^ Tregs, which are known to regulate Th1 and Th17 responses [28], have been discovered to secrete AREG and strongly contribute to tissue protection in LCMV infection [29]. Unexpectedly, we found that in *ICER* ^fl/fl^ *Foxp3* ^cre^ mice, a higher percentage of Tregs expressed TIGIT, indicating that while ICER‐KO Tregs appear to react to an inflammatory environment with the expression of TIGIT, they are impaired in effector function. PKA‐signalling via Gαs/cAMP attenuates AREG expression and wound‐healing phenotype [46]. A potential deregulation of this pathway in the absence of ICER could be responsible for the exacerbated tissue damage observed in the DSS colitis model. However, the exact mechanisms and a potential metabolic link remain to be studied.

In a tumour setting, increased PKA signalling following EP2/EP4 stimulation results in improved suppressive capacity by upregulation of surface molecules like CTLA‐4 and GITR, contributing to a tumour‐infiltrating Treg phenotype [47]. While we did not detect improved suppression in homeostasis or Th1‐like inflammation, the cancer microenvironment could present a viable avenue to study ICER‐dependent Treg function.

Overall, our findings suggest that ICER is a context‐dependent regulator of Treg function. Under homeostatic conditions, ICER is dispensable for Treg development and suppression of Teff proliferation. Under inflammatory conditions, however, ICER deficiency improved maintenance of Foxp3 expression but impaired control of Teff‐cell IFN‐γ and increased glycolytic activity. These results provide a framework for understanding how cAMP‐dependent transcriptional regulation shapes Treg function beyond canonical suppression and suggest that discrete metabolic programs may differentially control distinct Treg effector functions.

### 3.1 Data Limitations and Perspectives

One limitation of this study is the use of DSS colitis as the sole *in vivo* model to assess the consequences of ICER deletion in Tregs. Although this model was sufficient to reveal aggravated intestinal histopathology and impaired control of effector IFN‐γ  responses, it may not capture the full spectrum of Treg metabolic adaptation *in vivo*. In addition, while ICER deficiency was associated with enhanced glycolysis in Tregs, the molecular basis of this phenotype remains unresolved. Our data indicate that the increased glycolytic state is not fully explained by the mTOR‐related readouts examined here, but they do not yet establish the complete underlying mechanism. Thus, this study identifies ICER as a candidate regulator of Treg metabolic programming and inflammatory function, while the precise pathways linking ICER to metabolism and Treg effector control remain to be defined.

## 4 Methods

### 4.1 Animals

All mice were bred and maintained under specific pathogen‐free conditions at the animal facility of the Medical Center of Johannes Gutenberg University Mainz (Mainz, Germany) and Ribeirão Preto Medical School (Ribeirão Preto, Brazil). C57BL/6JRj mice were obtained from Janvier Labs and B6.SJL‐Ptprca Pepcb/BoyJ (CD45.1) mice were obtained from the Jackson Laboratory. ICER flox animals, described previously [22], were imported from the Institute of Infection Immunology TWINCORE—Centre for Experimental and Clinical Infection Research, Hannover, and crossed to B6129S‐Tg(Foxp3‐EGFP/icre)1aJbs/J (Foxp3cre) mice imported from Core Facility Laboratory Animal Services (CF‐LAS), Neuherberg. Full knockout mice were expanded from a spontaneous germline deletion event [24] and validated using primers: ICERko fw: 5’‐TGGAGCCTTGTTGAGACTG‐3’, ICERko rev: 5’‐ TGAAACTGTAGCCTAGTGGTG‐3’, ICERfl A fw: 5’‐GCCTTGTTGAGACTGTCATC‐3’, ICERfl A rev: 5’‐ATCTTCTCTCCCCTATCCCC‐3’, ICERfl B fw: 5’‐GCTTCCCCACAATTTAGCTC‐3’, ICERfl B rev: 5’‐TGTAGCCTAGTGGTGGTAAAC‐3’. All experiments were performed according to the guidelines of the Animal Welfare Committee of the Ribeirão Preto Medical School, University of São Paulo (protocol number: 1399/2024). For *in vitro* and *ex vivo* experiments, mice of both sexes were used in the age range of 7–20 weeks.

### 4.2 DSS Colitis

12–16‐week‐old male mice were offered water containing 2.5% DSS (MP Biomedicals) ad libitum for 6 days. Thereafter, animals were offered untreated water for an additional 6 days. Weight was measured daily. On the twelfth day, animals were sacrificed, and mLNs and colonic LP were prepared for flow cytometry analysis as well as for histology. Eight to twelve animals were used per group.

### 4.3 Histology

For haematoxylin and eosin staining, tissue samples were fixed in 4% formaldehyde (Carl Roth GmbH+Co.KG, Karlsruhe, Germany) and embedded in paraffin. Images were taken using a light microscope type Olympus BX51 (Olympus Corporation, Tokyo, Japan), and digitally using NDP.view2 (Hamamatsu) software. Scale bar: 250 µm for the detailed section and 200 µm for the overview section. Blinded histological scoring of colon sections was performed according to Erben et al. [48].

### 4.4 Lamina Propria Isolation

LP isolation was performed as described previously [49]. Briefly, colons or small intestines were isolated, Peyer's patches were removed, cut open longitudinally, and washed in ice‐cold PBS. Tissue was then incubated for 20 min in PBS with 30 mM EDTA and 1.5 mM DTT on ice, washed again, and incubated in PBS with 30 mM EDTA for 20 min at 37°C. Afterward, the tissue was washed in HBSS +calcium/+magnesium, minced, and digested with 0.5 mg/mL Collagenase D (Roche), 0.5 mg/mL DNase I (Applichem), and 0.04 mg/mL Dispase I (Corning) for 30 min at 37°C. Afterward, tissue was strained and resuspended in 40% Percoll. After, the suspension was overlaid on 80% and centrifuged for 20 min at 1200 x g. LP fraction at the interphase of the Percoll gradient was collected, washed, and stained for flow cytometry.

### 4.5 Murine T Cell Enrichment

Naïve T cells were isolated from spleen and peripheral lymph nodes using the EasySep Mouse Naive CD4^+^ T Cell Isolation Kit (Stemcell Technologies) according to the manufacturer's instructions. For sorting, the EasySep Mouse CD4^+^ T Cell Isolation Kit (Stem Cell) was used for pre‐enrichment and cells were stained with anti‐CD4 (RM4‐5; eBiosciences), anti‐CD25 (PC61.5; eBiosciences), anti‐CD44 (IM7; eBiosciences), and anti‐CD62L (MEL‐14; Tonbo Biosciences) antibodies and sorted using the Aria III (BD).

### 4.6 *In Vitro* T Cell Differentiation

Isolated naïve T cells were stimulated in RPMI (ThermoFisher) containing 10% FCS (Sigma), penicillin–streptomycin cocktail (ThermoFisher), and 50 µM β‐mercaptoethanol (Gibco). For iTreg differentiation, cells were stimulated with plate‐bound 5 µg/mL anti‐CD3 (BioLegend), soluble 1 µg/mL anti‐CD28 (BioLegend) in the presence of 200 U/mL rhIL‐2 (Peprotech) and 1 ng/mL rhTGF‐β (Peprotech) for 96 h. After 48 h, cells were treated with an additional 100 U/mL of rhIL‐2. For Th1 differentiation, cells were stimulated with plate‐bound 10 µg/mL anti‐CD3 (BioLegend), soluble 1 µg/mL anti‐CD28 (BioLegend) in the presence of 10 µg/mL anti‐IL‐4 (BioLegend) and 20 ng/mL IL‐12 (Peprotech) for 96 h. For Th0 differentiation, cells were stimulated with plate‐bound 10 µg/mL anti‐CD3 (BioLegend) and soluble 1 µg/mL anti‐CD28 (BioLegend) in the presence of 200 U/mL rhIL‐2 (Peprotech) for 96 h. For low‐glucose conditions, differentiation was performed using Seahorse XF RPMI Medium (Agilent) supplemented with glutamine and different concentrations of glucose.

### 4.7 nTreg Culture

nTregs were sorted as CD4^+^ Foxp3cre^+^ (GFP^+^) cells (Figure 1C,D) or CD4^+^ CD25^+^ CD127^low^ cells (Figure S3B) using anti‐CD4 (RM4‐5; eBiosciences), anti‐CD25 (PC61.5; eBiosciences), anti‐CD127 (A7R34; eBiosciences). Cells were then seeded at a density of 1 × 10^6^ cells/mL in 1 µg/mL anti‐CD3 and 5 µg/mL anti‐CD28 coated flat‐bottom plates in IMDM (ThermoFisher) containing 10% FCS, Penicillin–Streptomycin cocktail, 500 µM β‐mercaptoethanol and 1× nonessential amino acids (ThermoFisher). Starting on day 0, IL‐2 was replenished every 2 days. After 48 h, cells were transferred to uncoated wells. For stability assays, cells were additionally treated with 20 ng/mL rmIL‐12 (Peprotech) and 10 ng/mL rmIFN‐γ (Peprotech) every 2 days.

### 4.8 Flow Cytometry

For proliferation assays, T cells were stained before plating with 5 µM Cell Trace Violet (C34557) for 7 min at 37°C. For apoptosis measurement, cells were stained with Annexin V (1:200) (SKU 35‐6409‐T100) for 15 min at 37°C. PI (00‐6990‐50) was added to each sample immediately before acquisition on the cytometer. For all other stains, dead cells were excluded with the live/dead fixable dead cell stain kit (Life Technologies), and FcBlock was performed for 15 min at 4°C. For analysis of surface markers, cells were stained in PBS using the following fluorescence‐conjugated monoclonal antibodies: CD3e (145‐2C11; eBiosciences), CD45 (30‐F11; BioLegend), CD4 (GK1.5; eBiosciences), CD45.1 (A20; eBiosciences), CD45.2 (104; eBiosciences), CD39 (24DMS1; eBiosciences), TIGIT (GIGD7; eBiosciences), ICOS (7E.17G9; eBiosciences), and ST‐2 (U29‐93; BD). For intracellular staining of cytokine and transcription factors, cells were stained with IFN‐γ (XMG1.2; Tonbo biosciences), IL‐10 (JES5‐16E3; eBiosciences), IL‐17A (eBio17B7; eBiosciences), Foxp3 (FJK‐16s, eBiosciences), RORγt (B2D, eBiosciences), TNF‐α (MP6‐XT22; eBiosciences), CTLA‐4 (UC10‐4B9; eBiosciences), and Helios (22F6; BioLegend) using the Foxp3/transcription factor fixation/permeabilisation kit (eBiosciences) according to the manufacturer's instructions. For cytokine staining, cells were stimulated in vitro in the presence of phorbol‐12‐myristate‐13‐acetate (0.1 µg/mL; Sigma‐Aldrich) and ionomycin (1 µg/mL; Sigma‐Aldrich) for 2 h, followed by incubation for 2 h with brefeldin A (5 µg/mL; eBiosciences) at 1 × 10^6^ cells/mL before staining. Cells were acquired on a Cytoflex S (Beckman Coulter), and data were analysed with FlowJo software (v.10.8.1, Tree Star).

### 4.9 *In Vitro* Suppression Assay

Naïve CTV‐stained T cells obtained from Ly5.1 mice were seeded at a density of 5 × 10^5^/mL in complete RPMI in 96‐well round‐bottom plates. GM‐CSF DCs were generated from bone marrow of sex‐matched mice as described previously [50] and added at a density of 3 × 10^4^ cells/mL. To test their suppressive capacity, sorted nTregs or iTregs were added at different concentrations. Soluble anti‐CD3 was added at 1 µg/mL. For Figure S2, 10 µM NECA and for Figure 2, 10 ng/mL rmIL‐12 (Peprotech) were added at the beginning of co‐culture.

### 4.10 Seahorse

At 96 h of culture iTreg cells were harvested and plated on poly‐L‐lysine‐coated (Merck; 0.01% 4°C O/N) 96‐well XF cell culture microplates in XF assay medium (pH 7.4, both from Agilent) supplemented with 10 mM D‐glucose, 1 mM sodium‐pyruvate (Merck), and 2 mM L‐glutamine (Gibco) at a density of 15 × 10^4^ cells/well. Microplates were incubated for 45 min at 37°C in a non‐CO_2_ incubator and subjected to real‐time analysis of OCR using an XFe96 Extracellular Flux Analyzer (Agilent). For the mitochondrial stress test analysis, the XF mitochondrial stress test was performed according to the manufacturer's instructions, using subsequent injections of oligomycin (1 µM), FCCP (1.5 µM), and rotenone/antimycin A (0.5 µM). For glycolysis analysis, the XF Glycolytic Stress Test was performed according to the manufacturer's instructions, using subsequent injections of glucose (10 mM), oligomycin (1 µM), and 2‐DG (50 mM). For rapamycin treatment, Tregs were treated 24 h before analysis with 100 nM rapamycin.

### 4.11 RT‐qPCR

RNA was extracted as per the manufacturer's instructions using the Monarch Spin RNA isolation kit (NEB). Reverse transcription was performed according to the manufacturer's instructions using the ImProm‐II Reverse Transcription System (Promega). cDNA was used at 8 ng per reaction, and RT‐qPCR was performed using iQ SYBR Green Supermix (Bio‐Rad). The cycling program was 95°C for 120 s followed by 40 cycles of 95°C for 15 s and 60°C for 1 min. Each sample was assayed in triplicate, and the results were normalized to the level of housekeeping gene *Actb*. Primers used: ICER fw: 5’‐ATGGCTGTAACTGGAGATGAAACT‐3’, ICER rev: 5’‐GACTGTGCAGGCTTCCTGGTGAG‐3’, β‐Actin fw: 5’‐TGTTACCAACTGGGACGACA‐3’, β‐Actin rev: 5’‐GGGGTGTTGAAGGTCTCAAA‐3’.

### 4.12 Western Blot

For immunoblotting, SDS‐PAGE was performed on whole‐cell lysates. Proteins were then transferred to a PVDF membrane (Bio‐Rad) and incubated with primary antibodies overnight. For detection anti‐phospho (Ser2448) mTOR (Cell Signaling, 5536) diluted 1:200 and anti‐mTOR (Proteintech, 66888‐1‐Ig) diluted 1:200, anti‐rabbit IgG (Cell Signaling, 7074) diluted 1:10,000 and anti‐mouse IgG (Jackson ImmunoResearch Lab Inc., 115‐036‐062) diluted 1:10,000 were used. As a loading control for protein samples, anti‐GAPDH antibody (Cell Signaling, 5174) diluted 1:5000 was applied. ImageJ was used for quantification.

### 4.13 ELISA

The suppression assay supernatants were collected after 96 h of co‐culture and stored at −80°C until use. IL‐10 (R&D, #DY417) cytokine concentration was directly assessed by ELISA. Optical density (OD) was measured using a microplate reader (BioTek Cytation 5, Agilent), and the differential absorbance (ΔOD_450–570_) was recorded.

### 4.14 Statistical Analysis

Statistical analysis was performed with GraphPad Prism software (version 9; GraphPad Software Inc., San Diego, CA, USA). The *p*‐values were calculated using a Student's *t*‐test, one‐way ANOVA, or two‐way ANOVA, as indicated. Data are displayed as mean ± standard deviation (SD). A *p*‐value of < 0.05 was considered significant and marked by asterisks (*p< 0.05; **p < 0.01; ***p < 0.001). Data distribution was assumed to be normal, but this was not formally tested.

## Author Contributions

Study design by A.L.K., D.N., J.C.A.F., and T.S. Performance of experiments by A.L.K., L.S., D.N., G.B., S.O., H.C., F.B., and M.R. A.H. and M.G. performed the blinded analysis of the histological score. A.L.K. wrote the first draft of the manuscript. D.N., L.B., and T.S. revised the manuscript. J.C.A.F. and T.S. supervised the study. All authors contributed to the review and editing of the final manuscript.

## Ethics Statement

All animal experiments were approved by the Ethics Committee on Animal Use of Ribeirão Preto Medical School, University of São Paulo (protocol 1399/2024), and were conducted in accordance with institutional guidelines.

## Conflicts of Interest

The authors declare no conflicts of interest.

## Supporting information




**Supporting File**: eji70297‐sup‐0001‐SuppMat.docx.

## Data Availability

All data supporting the findings of this study are available from the corresponding author upon reasonable request.
