# Supplementary material for: Inducible cAMP Early Repressor Limits Glycolytic Programming in Regulatory T Cells and Supports Control of Inflammatory Colitis
Source: Eur J Immunol. 2026 Oct 14;56(10):e70297. doi: 10.1002/eji.70297 (PMC13669413; doi:10.1002/eji.70297)
Supplement: Supplementary file 1 [file EJI-56-e70297-s001.docx]

**Supplementary Figure 1. Treg differentiation is independent of ICER. (A)** Naive T cells and nTregs were sorted from SLOs of *Foxp3*^cre^, *ICER*^fl/fl^ *Foxp3*^cre^ and *ICER*^ko/ko^ mice. nTregs were lysed for RNA extraction. Naïve T cells were expanded for 96h under iTreg differentiating conditions and lysed for RNA extraction. qPCR was performed for detection of *Crem* P2 and P5 promoter transcript. **(B)** T cells were isolated from SLOs and lamina propria of SI were stained for flow cytometry analysis and cell number was calculated from total cell number and percentages of Foxp3^+^ cells. **(C)** Gating strategy of Foxp3 detection from SLOs. **(D)** Naïve T cells were isolated, expanded for 96h in iTreg differentiating conditions and stained for flow cytometry detection of Foxp3. **(E)** Single cell suspensions were generated from spleens of *ICER*^wt/wt^ and *ICER*^ko/ko^ mice and stained for flow cytometry analysis. **(F)** Gating strategy to detect Foxp3 in iTreg culture**. (G)** Suppression assay was harvested and stained for flow cytometry analysis of proliferating and IFN-γ-producing cells. Students t-test (**D, E**) and Two-way ANOVA with multiple comparisons (**B**) was used for statistical analysis. Mean ± SD, **p* < 0.05, ***p* < 0.01, ****p* < 0.001, ns, not significant

**Supplementary Figure 2. cAMP-mediated Treg suppression is independent of ICER. (A)** Naive T cells were sorted from SLO of *Foxp3*^cre^ mice and expanded under iTreg differentiating conditions for 48h or 96h in the presence or absence of 10 µM NECA. **(B)** CD45.1^+^ T cells were co-incubated with DCs and CD45.2^+^ WT or ICER-deficient Tregs for 96h and proliferation was assessed by flow cytometry. Representative histogram overlay of CTV of CD45.1^+^ T cells **(C)** Percentages of proliferating (CTVlow) CD45.1^+^ cells after 96h of incubation with WT Tregs (top) and ICER-deficient Tregs (bottom) different ratios of Treg:Teff in the presence or absence of NECA. **(D)** Naïve T cells isolated from *ICER*^wt/wt^ and *ICER*^ko/ko^ mice were expanded in Th1-polarising conditions for 96h and stained for flow cytometry analysis. Students t-test (**D**) and Two-way ANOVA with multiple comparisons (**C**) was used for statistical analysis. Mean ± SD, **p* < 0.05, ***p* < 0.01, ****p* < 0.001, ns, not significant

**Supplementary Figure 3. ICER affects Treg metabolism.** (**A**) Sorted Tregs from SLOs of *ICER*^fl/fl^ and *ICER*^fl/fl^ *Foxp3*^cre^ mice were stained with CTV, expanded for 96h and stained for flow cytometry analysis (**B+C**) Sorted Tregs and naïve T cells from SLOs of *ICER*^wt/wt^ or *ICER*^ko/ko^ mice were expanded for 96h in the presence of IL-2 or in iTreg polarising conditions respectively and stained with Annexin and PI to identify apoptotic cells by flow cytometry. (**D**) Representative oxygen consumption rate (OCR) of iTregs after 96h of differentiation. **(E)** Quantification of basal respiration, ATP production, maximal respiration, and SRC. (**F**) Sorted naïve T cells from SLOs of *ICER*^fl/fl^ and *ICER*^fl/fl^ *Foxp3*^cre^ mice were expanded in iTreg polarising conditions with decreasing concentrations of glucose in the media. After 96h cells were stained to assess survival by flow cytometry. (**G**) Western blot analysis of mTOR activity in WT and ICER-deficient iTregs after 96h of differentiation. Two-way ANOVA with multiple comparisons (**C, G**) was used for statistical analysis. Mean ± SD, **p* < 0.05, ***p* < 0.01, ****p* < 0.001, ns, not significant.

**Supplementary Figure 4. ICER deficient Tregs fail to contain IFN-γ production during DSS colitis.** (**A**) Colon length after 12 days of DSS colitis of *ICER*^fl/fl^ *Foxp3*^cre^ and *ICER*^fl/fl^ littermates. (**B**) Mesenteric lymph nodes were collected and lamina propria lymphocytes were isolated on day 12 of DSS colitis and analysed by flow cytometry. (**C**) Foxp3^+^ cell numbers were determined by flow cytometry and total cell number. (**D**) Frequencies of IL-10^+^ among Foxp3^+^ Tregs (**E**) Frequencies of Rorγt^+^ cells among Foxp3^+^ Tregs (**F**) Frequencies of IL-17A^+^ cells among Foxp3^+^ Tregs (**G**) Frequencies of IFN-γ^+^ cells among Foxp3^+^ Tregs (**H**) Frequencies of IL-17A^+^ cells among CD4^+^ Foxp3^-^ cells. Students t-test (**A**) and Two-way ANOVA with multiple comparisons (**C - H**) was used for statistical analysis. Mean ± SD, **p* < 0.05, ***p* < 0.01, ****p* < 0.001, ns, not significant.
